# Endothelial Nitric Oxide Synthase Gene T-786C Polymorphism in Renal Transplant Recipients

**Published:** 2011-05-01

**Authors:** N. Azarpira, B. Geramizadeh, S. Nikeghbalian, A. Bahador, R. Yaghobi, H. Karimi, M. Ayatolahi, M. H. Aghdai, H. Salahi, S. A. Malek-Hosseini, J. Roozbeh, M. Sagheb, G. H. Raisjalali, A. Behzadi

**Affiliations:** 1 *Shiraz Transplant Research Center, University of Medical Sciences, Shiraz, Iran*; 2 *Organ Transplant Center, Shiraz , University of Medical Sciences, Shiraz, Iran*

**Keywords:** Nitric oxide, Endothelial nitric oxide synthase, Polymorphism, Transplant, Acute rejection

## Abstract

Background: Nitric oxide (NO) is a major mediator in vascular biology, regulating regional blood flow. NO and the enzymes required for its production contribute to ischemia-reperfusion injury. The T-786C functional polymorphism in the promoter region substantially reduces promoter activity of the endothelial nitric oxide synthase (eNOS) gene and compromises endothelial NO synthesis.

Objective: To examine the association between T-786C (rs 2070744) single nucleotide polymorphism (SNP) in eNOS gene and the development of acute rejection in renal transplant patients.

Methods: 60 renal transplant recipients (30 with episodes of acute rejection (ARs) and 30 without rejection (non-ARs)), between June 2008 and March 2010, were included in this study. The polymorphism was determined by PCR-restriction fragment-length polymorphism analysis.

Results: The distribution of the genotypes were TT/TC/CC 60%, 33.4%, 6.6%, and 43%, 46.7%, 13.3% in ARs and non-ARs, respectively (p=0.28). The frequency of T-allele was 76.7% and 66.3%; and for C-allele was 66.6% and 33.3% in ARs and non-ARs, respectively (p=0.09). There were no significant associations between these polymorphisms and acute and chronic kidney allograft rejection.

Conclusion: We could not detect any significant association between polymorphism in T-786C of eNOS gene and the development of acute rejection.

## INTRODUCTION

Nitric oxide (NO) which is catalyzed by endothelial nitric oxide synthase (eNOS), plays an important role in the physiology of blood vessels. In the cardiovascular system, it causes vasodilatation, inhibition of platelet aggregation, prevention of leukocyte adhesion and suppression of vascular smooth muscle cell migration. Therefore, it may inhibit the initiation and/or progression of atherosclerosis [1]. NO has been shown to inhibit oxidative stress, cytokine release, leukocyte endothelial adhesion and apoptosis [2]. On a cellular-signaling level, the effects of NO include: inhibition of protein kinase C, activation of tyrosine kinase, inactivation of nuclear factor (NF)-κB and activation of G proteins [3]. NO is also an important mediator of ischemia-reperfusion injury (IRI). Decreased endogenous NO production resulting in capillary luminal narrowing is central in the pathogenesis of IRI [1]. Experimental inhibition of NO production is associated with increased risk of IRI [4-6]. In human studies, increased endothelial expression of eNOS after renal reperfusion is associated with good recovery from renal ischemia with good renal function [7]. Delayed graft function, a clinical indicator of ischemia-reperfusion injury, is strongly associated with acute rejection and long-term poor outcome [8, 9]. Therefore, genetic variation in eNOS may result in diminished availability of NO following IRI and subsequently affect renal function. The T-786C functional polymorphism in the promoter region is linked with decreased eNOS expression [10, 11]. This polymorphism has been reported to be associated with retinopathy in type 1 diabetes [12] and cardiovascular diseases such as hypertension and coronary arterial spasm [13, 14].

We conducted the present study to examine the prevalence of the *eNOS *T-786C polymorphism in a group of renal transplant recipients with and without acute rejection.

## MATERIALS AND METHODS

Sixty renal transplant recipients (30 with episodes of acute rejection (AR) and 30 without rejection (non-AR)), between June 2008 and March 2010, were selected for inclusion in this study. The Ethics Committee of Shiraz University of Medical Sciences approved this study. An informed written consent was obtained from all participants. Episodes of AR were recorded in post-transplant hospital course. An AR episode was defined based on clinical or biopsy findings according to Banff criteria [15]*.* “Clinical rejection” was defined as an increase in creatinine levels in the absence of infection, obstruction or evidence of drug toxicity. The clinical characteristics related to transplantation were retrieved from our Kidney Transplant Database. The routine immunosuppression regimen consisted of cyclosporine or tacrolimus, with mycophenolate mofetil (CellCept^®^) and prednisone. The daily dose of cyclosporine was then adjusted according to its blood concentration (C0); the target through concentration was 180 ng/mL. For the first line treatment of AR (either biopsy-proven or clinical), patients received methyl prednisolone pulse therapy. If there was no response to the initial anti-rejection therapy, antilymphocyte antibody (OK3) was used as the second line treatment. The serum creatinine level was used as a parameter for post-transplant graft function; the response was categorized by serum creatinine levels after anti-rejection therapy. 

Genotype analysis: 

Genomic DNA samples were prepared from leukocytes in whole blood and extracted using the commercial extraction kit (DNG plus DNA extraction kit, Cinagene Company, Tehran, Iran) according to the manufacturer’s instructions. The T-786C (rs 2070744) single nucleotide polymorphism (SNP) located at the promoter of *eNOS* gene, is recorded in the public dbSNP base [ 16 ]. This SNP was studied using a polymerase chain reaction (PCR)-restriction fragment length polymorphism (RFLP), according to Han Y, *et al* [ 17 ]. The PCR primers were (forward): 5’-CAG ATG ACA CAG AAC TAC AA-3’; and (reverse): 5’-GAG TCT GAC ATT AGG GTA TCC-3’.

The amplification condition was an initial denaturing cycle at 94 °C for 5 min, followed by 35 amplification cycles of 94 °C for 30 s, 55 °C for 30 s, 72 °C for 30 s to yield 338-bp products. After incubation with *MboI* restriction enzyme (Fermentase, Litevany), we found 338-, 195- and 143-bp fragments for the CT heterozygotes; a single 338-bp product for the TT homozygotes, and 195- and 143-bp products for the CC homozygotes.

Statistical Analysis:

Differences in gender, immunosuppression, etiology and *NOS *T-786C genotype distributions between AR and non-AR were analyzed by χ^2^ test. Differences in age were analyzed using *Student’s* *t* test. The level of significance was set at p<0.05. Analyses were performed with SPSS^®^ for Windows^®^ ver 15 (SSPS Inc, Chicago, IL, USA).

## RESULTS

We compared the *eNOS* T-786C polymorphism in a group of kidney allograft with and without AR. Patients included 43 men and 17 women with a mean±SD age of 32.1 + 11.1 years. The detailed data of patients’ demographic characteristics and pre-transplant status are shown in Table 1. Statistical analysis of recipient demographic characteristics including donor and recipient age/gender and their primary underlying kidney disease showed no statistically differences between the ARs and non-ARs. The majority of organs were donated from cadavers.

**Table 1:** Demographics of kidney graft recipients

**Parameter**	**ARs (%)**	**Non-ARs (%)**
Number of patients	30 (100)	30 (100)
Recipient gender		
Male Female	18 (60) 12 (40)	25 (83) 5 (17)
Mean±SD recipient age (yrs)	31.45±8.9	32.1±10.1
Mean±SD donor age (yrs)	27.9±10.3	29.44±14.1
Donor gender		
Male Female	21 (70) 7 (30)	18 (60) 12 (40)
Primary disease		
End-stage renal disease Diabetic nephropathy Glumerulonephritis	24 (80) 3 (10) 3 (10)	28 (94) 1 (3) 1 (3)
Living donor Deceased donor	10 (30) 20 (70)	6 (20) 24 (80)
Immunosuppression CSA + CellCept + prednisone Tacrolimus + CellCept + prednisone	5 (17) 25 (83)	28 (94) 2 (6)
Histological grade of rejection		
Ia Ib IIa IIb III	20 (70) 8 (24) 1 (3) 1 (3) 0 (0)	

ARs: Acute rejection

CSA: Cyclosporine

The distribution of genotypes and allele frequencies between groups is shown in Tables 2 and 3; we did not observe any significant differences. The distribution of the genotypes were TT/TC/CC 60%, 33.4%, 6.6%, and 43%, 46.7%, 13.3% in ARs and non-ARs, respectively (p=0.28). The frequency of T-allele was 76.7% and 66.3%; and for C-allele was 66.6% and 33.3% in ARs and non-ARs, respectively (p=0.09). This polymorphism did not influence short-term renal allograft outcome. When the study sample was categorized into two groups of homozygous TT and others (TC & CC**),** the comparison between the two groups was still not significant; therefore, we could not find any association between the *eNOS *T-786C polymorphism and acute allograft rejection.

**Table 2:** Distribution of nitric oxide -786T>C (rs 2070744) genotypes in acute rejection (ARs), non-ARs.

HLA-G Genotype	ARs (%) (n=30)	non-ARs (%) (n=30)	p value
TT TC CC	18 (60) 10 (33.4) 2 (6.6)	12 (43) 14 (46.7) 4 (13.3)	0.28
TT TC & CC	18 (60) 12 (40)	12 (43) 18(57)	0.08

**Table 3:** Distribution of nitric oxide -786T>C alleles in acute rejection (ARs) and non- ARs.

**Allele**	**ARs (%)**	**Non-ARs (%)**	***p*** ** value**
**T** **C**	46 (76.7) 14 (23.3)	40 (66.6) 54 (33.3)	0.09

## DISCUSSION

Renal transplantation is the treatment of choice for patients with end-stage renal disease. However, episodes of AR have a negative impact on short- and long-term graft outcome. The incidence of AR is 10%–30% in the first year after transplant, especially the first month post-transplant [18].

NO is an endogenous immunomodulator which is involved in immunoregulation and host defense mechanisms. It inhibits platelet aggregation and platelet and leukocyte adhesion to vascular endothelium and also has anti-proliferative effects on vascular smooth muscle cells [19]. There are reports about the role of NO in IRI and allograft rejection in liver, heart; kidney and pancreatic islet cell transplant [20-26]. Inhaled NO or NO donor drugs are novel treatments that have been used clinically to diminish IRI.

The inducible NOS isoform (iNOS) is expressed in glomerular mesangial cells, proximal tubules and distal tubular segments in kidneys [27-29]. Significant increase in serum NO levels was reported during episodes of AR in renal transplant recipients [26]. The iNOS expression is increased after stimulation by infiltrating cells in AR of renal allograft as well as presence of inflammatory cytokines [23, 27, 29, 30].

NO inhibits *in vitro* neutrophil adhesion to endothelium by blocking the nuclear factor (NF)-κB-dependent transcription of adhesion molecules [6]. 

Infections and surgical stress had an important role in stimulating NO production after transplant, while drugs such as glucocorticoids or calcineurin inhibitors such as tacrolimus inhibit its production [24].

Therefore, NO could increase in AR as a response to various cytokines that participate in the process of rejection. Moreover, there are known factors that can influence NO production in a transplant patient. NO is rapidly degraded to the stable end-products *e.g.*, nitrite and nitrate which can be measured in serum and urine [31, 32].

The genetic variations in the endothelial NO synthase gene may influence serum NO levels and has effect on the inflammatory process. The -786T>C polymorphism has been associated with significant variation in eNOS promoter activity [11]. 

In this study no association was found between genotypes or allele frequency of this SNP in renal transplant recipients who experienced AR *vs* recipients without rejection.

Yilmaz, *et al*, studied on G894T mutation at exon 7 of the eNOS gene and correlation with chronic allograft nephropathy. This polymorphism did not influence long-term renal allograft outcome and is not considered as a risk factor for chronic allograft failure [33]. Viklický, *et al*, compared the eNOS (G894T) gene polymorphism in patients with preserved graft function over 15 years and in a control group of transplant recipients. They mentioned no differences in allele and genotype distributions between the studied groups. There were no links between genotypes, renal function and atherosclerosis risk factors in these patients [34]. Sezer, *et al*, studied on angiotensin II type 1 receptor (ATR1) and eNOS gene polymorphism in renal transplant patients. They found that bb allele of the eNOS and non-AA allele of ATR1 1166 gene were associated with an anti-inflammatory state and may predict renal outcome in transplant patients [35].

In this study, we could not show any significant association between polymorphism in T-786C of eNOS gene and short-term renal graft function.
